# QTL-By-Environment Interaction in the Response of Maize Root and Shoot Traits to Different Water Regimes

**DOI:** 10.3389/fpls.2018.00229

**Published:** 2018-02-23

**Authors:** Pengcheng Li, Yingying Zhang, Shuangyi Yin, Pengfei Zhu, Ting Pan, Yang Xu, Jieyu Wang, Derong Hao, Huimin Fang, Chenwu Xu, Zefeng Yang

**Affiliations:** ^1^Jiangsu Key Laboratory of Crop Genetics and Physiology, Co-Innovation Center for Modern Production Technology of Grain Crops, Key Laboratory of Plant Functional Genomics of the Ministry of Education, Yangzhou University, Yangzhou, China; ^2^Nantong Key Laboratory for Exploitation of Crop Genetic Resources and Molecular Breeding, Jiangsu Yanjiang Institute of Agricultural Sciences, Nantong, China

**Keywords:** maize, root system architecture, QTL-by-environment interaction, root plasticity, drought, QTL mapping

## Abstract

Drought is a major abiotic stress factor limiting maize production, and elucidating the genetic control of root system architecture and plasticity to water-deficit stress is a crucial problem to improve drought adaptability. In this study, 13 root and shoot traits and genetic plasticity were evaluated in a recombinant inbred line (RIL) population under well-watered (WW) and water stress (WS) conditions. Significant phenotypic variation was observed for all observed traits both under WW and WS conditions. Most of the measured traits showed significant genotype–environment interaction (GEI) in both environments. Strong correlations were observed among traits in the same class. Multi-environment (ME) and multi-trait (MT) QTL analyses were conducted for all observed traits. A total of 48 QTLs were identified by ME, including 15 QTLs associated with 9 traits showing significant QTL-by-Environment interactions (QEI). QTLs associated with crown root angle (*CRA2*) and crown root length (*CRL1*) were identified as having antagonistic pleiotropic effects, while 13 other QTLs showed signs of conditional neutrality (CN), including 9 and 4 QTLs detected under WW and WS conditions, respectively. MT analysis identified 14 pleiotropic QTLs for 13 traits, SNP20 (1@79.2 cM) was associated with the length of crown root (CR), primary root (PR), and seminal root (SR) and might contribute to increases in root length under WS condition. Taken together, these findings contribute to our understanding of the phenotypic and genotypic patterns of root plasticity in response to water deficiency, which will be useful to improve drought tolerance in maize.

## Introduction

Maize (*Zea mays* L.) is the most widely grown staple food, feed, and industrial crop, and it plays a critical role in supporting the growing world population. Although maize has become one of the most productive crops after intensive improvement, the yield of maize is frequently limited by various biotic and abiotic stress factors, such as drought, salinity, high and low temperatures, nutrient deficiencies, disease, and insect pests. Of these stresses, maize is most susceptible to drought (Araus et al., 2012). To cope with the negative effects of drought, maize has developed various adaptive strategies. Extensive research into the responses of maize to drought stress has focused on identified key secondary traits, such as anthesis-to-silking interval (ASI), leaf area, extent of leaf rolling, osmotic adjustment, stomatal conductance, canopy temperature, and ABA concentration (Bruce et al., 2002; Messmer et al., 2009; Li et al., 2015b). Roots are the essential organ for perceiving water deficit signals and water uptake (Meister et al., 2014). In recent years, several studies have demonstrated that ideal root phenotypes, such as root growth angle and number, can improve water acquisition from dry soil (Lynch and Wojciechowski, 2015; Gao and Lynch, 2016). Dissecting the genetic basis of such traits are essential for the development of more drought-tolerant maize cultivars.

The maize root system consists of a primary root (PR), seminal roots (SR), several whorls of crown root (CR), and lateral roots (LR; Hochholdinger et al., 2004). The PR is the first root to emerge from the seed at germination (Salvi et al., 2016). Seminal roots are present in the ungerminated caryopsis and emerge between the scutellum and the first internode. Both the PR and SRs are important for the early development of the seedling in the first 2 weeks following germination (Salvi et al., 2016). Crown roots arise from the basal intercalary meristem of the lower internodes of the stem. Crown roots are major structural components of the maize root system that play important roles in anchorage and soil resource acquisition during vegetative growth and reproductive development (Gao and Lynch, 2016). Deeper roots are an important strategy to access water stored deep in the soil, and the utility of rooting depth for drought tolerance is well-documented in maize, rice, and wheat (Wasson et al., 2012; Lynch, 2013; Uga et al., 2013). Root angle is widely recognized to play an important role in determining rooting depth, and steep growth angles are superior for water acquisition under drought (Mace et al., 2012; Lynch, 2013; Ali et al., 2015). In cereal crops, deeper rooting is achieved by a combination of the root growth angle, root length, root diameter, and root number (Lynch, 2013; Ali et al., 2015). The root growth angle determines the direction of root elongation (Ali et al., 2015), larger root diameter can increase the ability of the root to penetrate deeper soil strata (Lynch, 2013), and low CRNs in maize can improve drought tolerance by increasing rooting depth (Gao and Lynch, 2016). Increased root biomass, root length density, and rooting depth are often considered to be primary drivers of drought avoidance (Kashiwagi et al., 2005). In addition, root architecture also shows high plasticity in response to the heterogeneous distribution of soil resources (Yu et al., 2014; Li et al., 2016). Multiple studies have reported that plasticity in certain root traits can improve plant performance under stress (Trachsel et al., 2013; Tran et al., 2015; Sandhu et al., 2016). A better understanding of root functional traits and the plasticity to water availability is essential to increase crop productivity under drought conditions.

Although root architectural traits have potential for breeding more drought-tolerant maize varieties, selection for optimal root systems is not routine due to the difficulty in directly accessing root traits (Cai et al., 2012; Burton et al., 2014). Marker-assisted selection (MAS) is a promising breeding strategy for improving complex traits. Mapping QTLs for root architectural traits would help to improve drought adaptation via MAS. In maize, many QTLs that regulate root system traits have been identified in several linkage populations, including root diameter (Burton et al., 2014), root number (Cai et al., 2012; Ku et al., 2012), root length (Hund et al., 2011), and root angle (Omori and Mano, 2007). Several root QTLs have also been mapped in other cereal crops. Taking root angle as an example, six major QTLs (*DRO1, DRO2, DRO3, DRO4, DRO5*, and *qSOR1*) for root angle have been identified in rice (Uga et al., 2015), and *DRO1* is the first cloned gene associated with deep root that can improve the ability to avoid drought (Uga et al., 2013). The identification and cloning of favorable loci for root growth have been used to improve drought resistance in maize (Giuliani et al., 2005) and rice (Uga et al., 2013). However, the genetic basis of root plasticity to water availability has not been fully elucidated in maize.

In the present study, a maize recombinant inbred line (RIL) population derived from a cross of two parental lines, DH1M and T877, was evaluated under water stress (WS) and well-watered (WW) conditions. Differences in the root system in response to different soil water condition was evaluated in controlled environments, and multi-trait (MT) and multi-environment (ME) QTL mapping for root traits in different water regimes was conducted. Our objectives were as follows: (i) investigate the key features of root plasticity related to water availability; and (ii) identify main QTLs, QTL-by-Environment Interaction (QEI) and pleiotropic QTLs for root traits.

## Materials and methods

### Plant materials and growth conditions

The RIL population consisting of 204 F8 lines was derived from a cross between two inbred lines, DH1M (female parent) and T877 (male parent) using single-seed descent. Plants were grown in a greenhouse located on the campus of Yangzhou University. The experiment used a completely randomized design with three replicates per line. Maize seeds of 204 lines were sterilized for 20 min in a 10% solution of H_2_O_2_, washed with distilled water, soaked in saturated CaSO_4_ for 6 h, and then germinated in the dark on moist filter paper at 28°C in a germination chamber for 2 days. Three seedlings of uniform size from each line were transplanted to round black plastic pots (18 cm in depth, 20 cm in diameter). Each pot was filled with 3 L light nutritional soil, and 2 days before planting, all pots were watered to saturation with deionized water. In the first 7 days, all pots received 100 mL of deionized water every 2 days. Then, 200 mL of deionized water used to irrigate plants in the WW treatment group every 4 days, and the WS treatment plants received no further irrigation until harvest. Seven days after sowing, seedlings were thinned to one per pot.

### Plant phenotyping

The seedlings were harvested at 35 days after sowing. Plant height (PH, cm) was measured from the coleoptilar node to the tip of the longest leaf. The third leaf (unfolded leaf) was selected for measuring leaf length (LL, cm), leaf width (LW, cm), and chlorophyll content (SPAD). SPAD was measured using a SPAD-502 PLUS chlorophyll meter (Minolta, Japan). Total shoot fresh weight (SFW, g) and dry weight (SDW, g) were measured. Shoot dry weights were determined after oven-drying at 70°C to a constant weight, and the shoot dry weight was measured using an electronic balance. Roots were separated from the soil by vigorous rinsing through a 2-mm sieve. Roots were stored at −20°C before the measurements. The length of primary root (PRL, cm), seminal root (SRL, cm), and crown root (CRL, cm) was measured with a ruler. The number of seminal roots (SRN) and crown roots (CRN) was counted. Three roots on third whorl were selected to measure crown root angle (CRA) and diameter (CRD, mm). Root angles were measured with a protractor as degrees from horizontal: Horizontal roots were classified as 0°, vertical roots as 90° (Trachsel et al., 2013). Selected crown roots were cut off, and a Vernier caliper was used to measure root diameter.

### Genotyping and construction of genetic linkage maps

All RILs and parental inbred lines were genotyped using an Affymetrix microarray CGMB50K SNP Array containing 56,000 maize SNPs at China Golden Marker (Beijing) Biotech, China. The cosegregating SNP markers were considered as belonging to the same recombination bin using a home-made Perl script. All called bins were used to construct the genetic linkage map using JoinMap version 4.0 software (Van Ooijen, 2006), with the Kosambi mapping function used to calculate the genetic distance between markers. The genotype and phenotype data can be downloaded from the website https://pan.baidu.com/s/1geDpnJt.

### Data analysis

The R software package (R Development Core Team, 2013) was used to perform statistical analyses on the raw data from each experiment. Analysis of variance (ANOVA) was used to test for significant differences between treatments, lines, and interactions (genotype–environment interaction, GEI). Mean values of each line across the two experiments were used for subsequent phenotypic summarization, correlation analysis, principal component analysis (PCA), and QTL mapping. PCA was performed using the “prcomp” function in R, with further visualization performed using the “ggfortify” package. The “lme4” package was used to estimate genotypic variance ($\sigma_{G}^{2}$), G–E interaction variance ($\sigma_{G}\times {\text{E}}^{2}$), and error variance ($\sigma_{e}^{2}$). The broad-sense heritability (*h*^2^) of each measured trait was calculated as previously described by Hallauer and Miranda (1981).

QTL mapping based on data for root and shoot traits under WW and WS conditions was conducted using ME analysis and a MT approach to detect main QTLs, GEI and pleiotropic QTLs with the QTL library in GenStat for Windows v18 (VSN International, Hemel Hempstead, UK). A step size of 10 cM, a minimum cofactor proximity of 30 cM, a minimum separation between selected QTLs of 20 cM, and a genome-wide significance level of *P* < 0.05 were used for the QTL analysis. The whole genome was first scanned using simple interval mapping (SIM) with selected cofactors for two rounds of composite interval mapping (CIM; Malosetti et al., 2004; Boer et al., 2007). A final QTL model was selected by backward selection of the selected cofactors, with the allelic effect of each QTL estimated in each environment (ME) or each trait (MT).

## Results

### Phenotypic variation in root traits and shoot traits under WW and WS conditions

**Figure 1:**
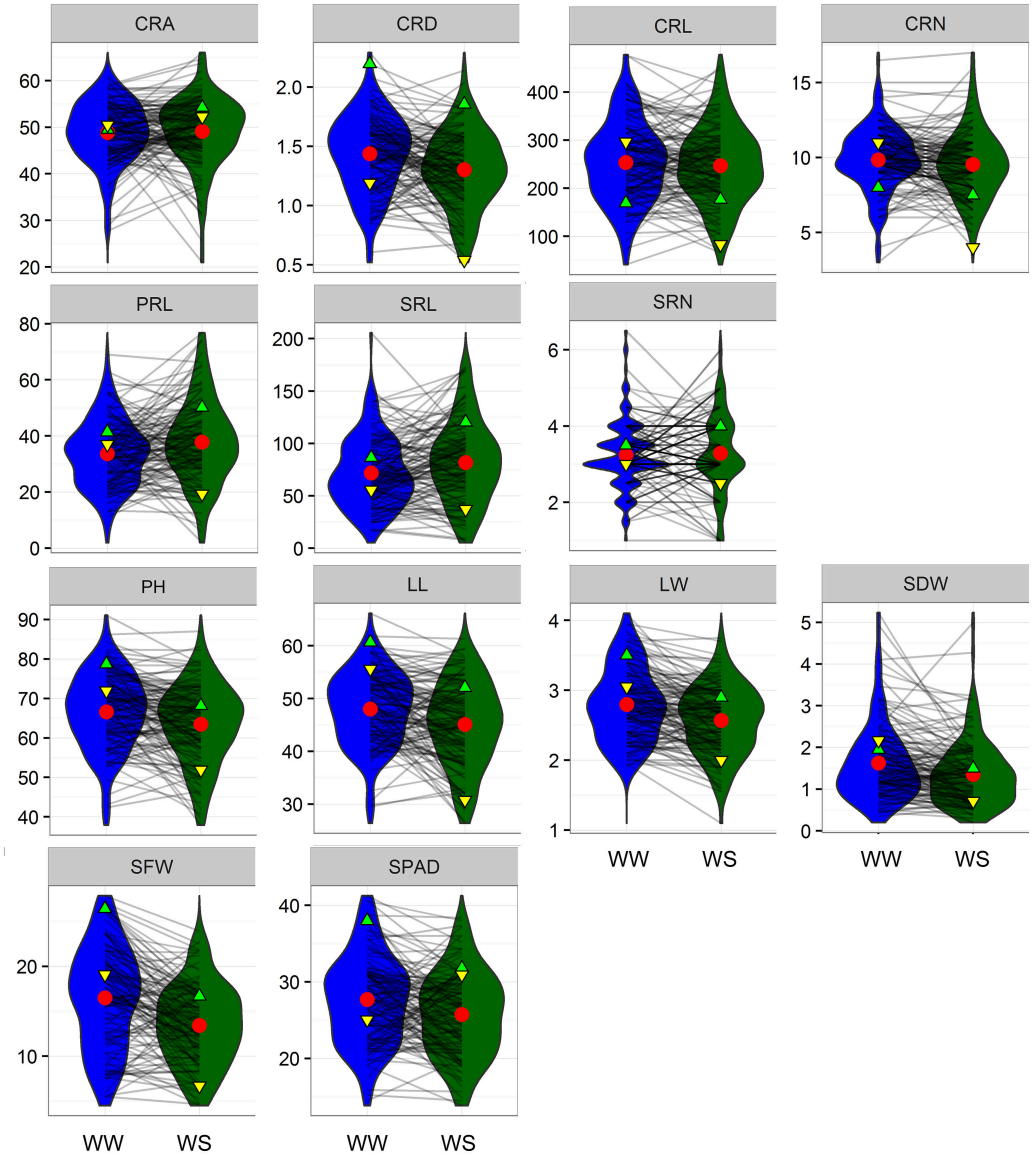
Reaction norms and phenotypic variation of root traits under well-watered (WW) and water stress (WS) conditions. Values of parental lines DH1M and T877 are indicated by green and yellow triangles, respectively. Red circles represent population mean values. CRA, crown root angle; CRD, crown root diameter; CRL, crown root length; CRN, crown root number; PRL, primary root length; SRL, seminar root length; SRN, seminar root number; PH, plant height; LL, leaf length; LW, leaf width; SDW, shoot dry weight; SFW, shoot fresh weight; SPAD, chlorophyll content measured by SPAD-502 PLUS chlorophyll meter.

Maize plants were grown under WW and water-stressed (WS) conditions in a greenhouse, and seven root traits, including crown root (CRA, CRD, CRL, and CRN), PRL, and seminal root number (SRL and SRN), and six shoot traits, including plant height (PH), leaf traits (LL and LW), shoot biomass (SDW and SFW), and SPAD, were evaluated three times. Water stress decreased CRD, CRL, CRN, PH, LL, LW, SDW, SFW, and SPAD and increased CRA, PRL, and SRL (Figure 1, Table 1). Water stress had the greatest influence on SFW and SPAD; the values for these parameters were reduced by 16.62 and 17.96%, respectively. Frequency distributions of the different traits in the RIL population showed that all variables exhibited normal distributions under WW and WS conditions (Figure 1). Coefficients of variation ranged from 14.0 to 56.7% under WW conditions and 15.43–57.42% under WS conditions, indicating the existence of considerable phenotypic variation (Table 1). Genotypic variation was significant for all investigated traits at *P* < 0.001, and 12 traits showed significant variation under different water regimes, except SRN. CRA, CRD, CRL, PRL, SRL, LL, SDW, SFW, and SPAD showed significant G × E interaction effects (Table 1). The heritability (*h*^2^) of root-related traits were moderate, ranging from 30.4% (CRA) to 86.6% (CRL) under WW and from 37.9 to 74.0% under WS conditions. The heritability (*h*^2^) of shoot-related traits was rather high, varying from 69.5 to 87.4% under WW and from 78.7 to 83.3% under WS condition (Table 1).

**Table 1:** Statistics of the analyzed traits in RIL population under well-watered (WW) and water stress (WS) conditions.

**Trait**	**Treatment**	**RIL population**						**ANOVA**		
		**Means**	**Min**	**Max**	**CV**	***h^2^* (%)^a^**	**Stress effect^b^**	**RILs**	**Treatment**	**G × E**
CRA	ww	47.33	24.50	61.00	14.00	30.4	3.74	^**^^c^	^**^	^*^
	ws	49.17	21.00	66.00	15.43	37.9				
CRD	ww	1.44	0.60	2.29	21.57	64.9	−9.01	^**^	^**^	^*^
	ws	1.31	0.54	2.14	24.35	62.1				
CRL	ww	253.34	41.00	477.50	33.00	86.6	−2.24	^**^	^*^	^*^
	ws	247.67	65.50	455.30	32.29	74.0				
CRN	ww	9.85	3.00	16.50	21.75	73.3	−3.29	^**^	^*^	NS
	ws	9.53	4.00	17.00	23.54	63.9				
PRL	ww	33.43	3.70	69.00	35.34	42.6	14.00	^**^	^**^	^*^
	ws	38.11	5.60	76.70	37.72	51.5				
SRL	ww	71.90	16.40	205.45	45.47	70.5	13.22	^**^	^**^	^*^
	ws	81.41	5.20	173.15	47.56	66.3				
SRN	ww	3.24	1.00	6.50	27.22	47.9	2.01	^**^	NS	NS
	ws	3.30	1.00	6.00	30.96	68.7				
PH	ww	66.51	39.00	91.05	14.38	77.5	−4.52	^**^	^**^	NS
	ws	63.51	37.90	87.05	15.51	82.5				
LL	ww	47.89	27.40	66.15	14.44	75.4	−5.74	^**^	^**^	^*^
	ws	45.14	26.40	61.30	17.46	79.5				
LW	ww	2.79	1.85	4.10	18.71	83.0	−3.13	^**^	^**^	NS
	ws	2.57	1.10	3.75	19.01	78.7				
SDW	ww	1.62	0.30	5.23	56.70	87.4	−7.93	^**^	^**^	^*^
	ws	1.35	0.20	5.00	57.42	83.3				
SFW	ww	16.41	4.51	27.88	34.10	69.5	−16.62	^**^	^**^	^*^
	ws	13.46	4.57	23.95	32.55	79.4				
SPAD	ww	27.64	14.45	41.25	20.68	73.1	−17.96	^**^	^**^	^*^
	ws	25.66	13.85	38.40	21.38	79.7				

a *h^2^(%), broad-sense heritability*.

b *Stress effect = (ws–ww)/ww × 100%*.

c Highly significant effect is indicated by

“**” (P < 0.01), significant effect is indicated by

“*” *(P < 0.05), “NS” indicated the effect was not significant*.

**Figure 2:**
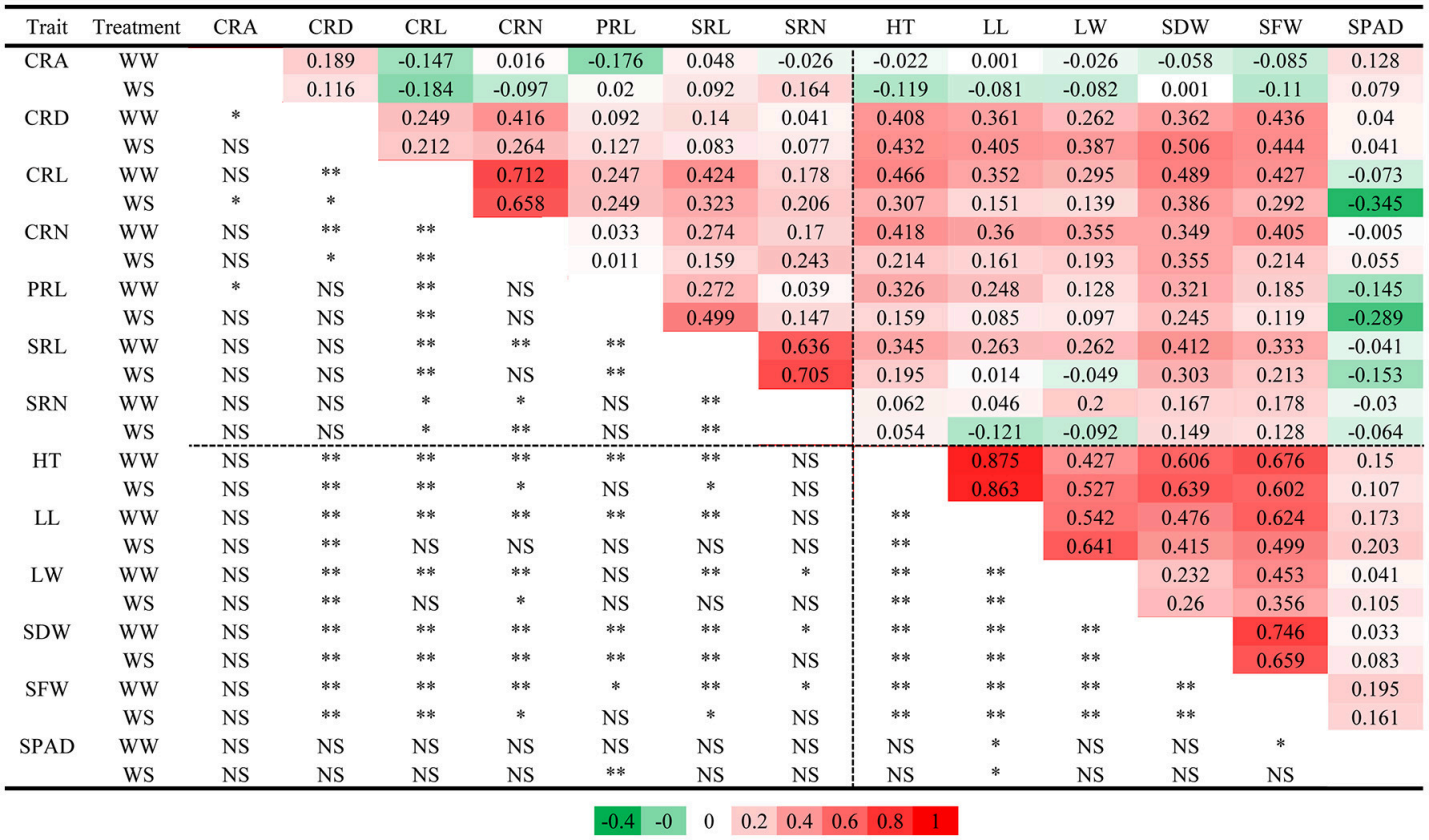
Pearson correlation coefficients for 13 measured traits in RIL population. **(Upper right)** Pearson correlation coefficients; **(Lower left)** Pearson correlation test, highly significant correlation between traits is indicated by “^**^” (*P* < 0.01), significant correlation between traits is indicated by “^*^” (*P* < 0.01), “NS” indicated the correlation was not significant.

**Figure 3:**
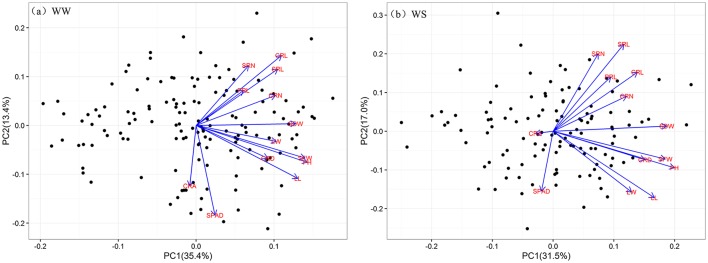
Principal components analysis of traits under WW and WS conditions. The projection of 13 traits onto the first and second principal components. Black dots indicate the position of the RILs as determined by their values on the given principal component. Blue lines represent vectors that quantify the magnitude and direction of a trait's contribution to the principal component under WW **(a)** and WS **(b)** conditions.

Regardless of different water regimes, CRA showed no significant correlation with other traits, except a very small correlation with CRD (WW, *r* = 0.189), CRL (WS, *r* = −0.184), and PRL (WW, *r* = −0.176). CRD was significantly correlated with shoot traits under WW (*r* = 0.262–0.436) and WS (*r* = 0.387–0.506), but SPAD was not (Figure 2). As expected, all shoot-related traits measured under WW and WS conditions showed a positive correlation (*r* = 0.260–0.875), except for SPAD. In general, the water treatment had little effect on the correlation coefficient between traits, indicating that the root traits and shoot traits synergistically respond to WS. Principal component analyses (PCA) were conducted among the RILs under WW and WS conditions (Figures 3A,B). The first two major principal components (PC1 and PC2) are projected, respectively, onto x- and y-axes in the figure. The first component (PC1) represented more than 30% of the variability under both WW and WS conditions and accounted primarily for most traits except for SPAD and CRA. PC2 explained 13.4% variation and accounted primarily for SPAD and CRA under WW, while 17% variation mainly accounted for SPAD under WS (Figure 3).

### Genetic map construction

**Figure 4:**
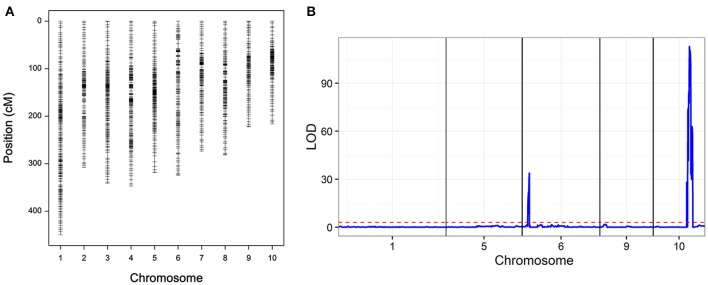
**(A)** Genetic map of recombinant inbred line (RIL) population and **(B)** quantitative trait loci (QTL) mapping of leaf sheath color.

The RIL populations were genotyped using the CGMB50K SNP Array, and a total of 56,000 SNPs were detected. After quality control, 9,780 high-quality polymorphic SNPs were used to construct linkage maps. The co-separation markers were considered as recombination bin, resulting in a map with 1,868 marker bins (Figure 4A, Table S1). The total length of the linkage map was 3,081.8 cM with an average interval of 1.65 cM, the largest interval on the linkage map was 12.85 cM. To examine the quality of the genetic map, QTL mapping was conducted for leaf sheath color, which has high heritability. Two QTLs were identified, the largest one located on chromosome 10 with a peak at 150.7 Mb (LOD = 112.9; Figure 2C). The cloned *r1* (colored1) gene was located in the confidence interval of this QTL (Figure 4B).

### Multi-environment and multi-trait QTL analysis

To identify the genetic basis of maize root plasticity to water availability, we conducted QTL mapping in the RIL population using a ME approach. In total, 48 QTLs were mapped for the thirteen analyzed traits under WW and WS environments (Table 2). A total of 15 QTLs, associated with 5 root traits (CRA, CRL, CRN, PRL, and SRL) and 4 shoot traits including LL, LW, SFW, and SPAD, showed significant Q × E interactions, of which two QTLs (*CRA2* and *CRL1*) had antagonistic pleiotropic effects in the two different environments, other QTLs with QEI were only identified in one of the conditions. The phenotypic effects of four QTLs: *CRN1, PRL3, SFW3*, and *SPAD2* were 26-, 9.72-, 13.01-, and 23.38-times higher, respectively, in one environment than the other (Table 2). Of the identified QTLs, 28 and 11 possessed a favorable allele from T877 and DH1M under both conditions (Table 2).

**Table 2:** QTLs for the analyzed traits in RIL population detected by the multi-environment analysis approach under well-watered (WW) and water stress (WS) conditions.

**Trait**	**QTL**					**WW**			**WS**			**G × E^b^**	**Ratio^c^**
	**Name**	**Chr**.	**Pos**.	**Marker**	**-log10P**	***r*^2^**	***P***	**Effect^a^**	***r*^2^**	***P***	**Effect**		
CRA	*CRA1*	1	152.1	SNP62	3.02	3.6	0.001	1.43	4.2	0.001	1.43	No	
	*CRA2*	1	401	SNP251	2.95	3.4	0.016	1.40	4.2	0.013	−1.44	Yes	−1.03
	*CRA3*	6	210.7	SNP1198	2.43	2.6	0.004	1.23	3.1	0.004	1.23	No	
	*CRA4*	9	191.5	SNP1711	2.81	3.3	0.002	1.38	3.9	0.002	1.38	No	
CRD	*CRD1*	2	289.6	SNP414	2.05	3.0	0.009	0.05	2.7	0.009	0.05	No	
CRL	*CRL1*	1	76.9	SNP19	1.70	0.1	0.020	−2.24	3.8	0.020	14.39	Yes	−6.42
	*CRL2*	1	449.8	SNP264	2.63	3.7	0.002	−15.84	4.6	0.002	−15.84	No	
	*CRL3*	7	121.9	SNP1347	3.52	5.1	0.000	18.74	6.4	0.000	18.74	No	
	*CRL4*	8	18.2	SNP1418	2.24	3.0	0.006	14.42	3.8	0.006	14.42	No	
CRN	*CRN1*	2	178.1	SNP372	3.06	8.0	0.000	0.60	0.0	0.891	0.02	Yes	26.0
	*CRN2*	7	123.7	SNP1349	2.87	8.0	0.000	0.60	0.2	0.596	0.09	Yes	6.71
PRL	*PRL1*	4	139	SNP712	2.11	4.1	0.008	2.56	3.7	0.008	2.56	No	
	*PRL2*	6	22.5	SNP1083	1.81	3.6	0.023	2.39	0.8	0.258	−1.19	Yes	−2.02
	*PRL3*	10	36.3	SNP1725	3.42	9.3	0.000	3.85	0.1	0.702	0.40	Yes	9.72
SRL	*SRL1*	1	389.8	SNP246	2.28	2.9	0.005	−5.50	2.3	0.005	−5.50	No	
	*SRL2*	2	187.8	SNP374	5.58	14.4	0.000	12.26	1.2	0.097	4.02	Yes	3.05
	*SRL3*	3	69.1	SNP433	4.23	6.9	0.000	8.53	5.6	0.000	8.53	No	
	*SRL4*	3	197.6	SNP571	3.42	5.1	0.000	7.29	4.1	0.000	7.29	No	
	*SRL5*	4	3.1	SNP636	2.83	3.8	0.001	6.33	3.1	0.001	6.33	No	
	*SRL6*	4	198.1	SNP773	2.94	5.1	0.001	−7.30	4.1	0.001	−7.30	No	
	*SRL7*	4	275.5	SNP830	2.68	1.2	0.158	−3.58	3.1	0.013	6.34	Yes	−1.77
	*SRL8*	7	0	SNP1239	3.83	5.5	0.000	7.60	4.4	0.000	7.60	No	
	*SRL9*	10	48.6	SNP1730	4.16	0.2	0.549	1.45	6.9	0.000	9.46	Yes	6.52
SRN	*SRN1*	3	70.4	SNP434	5.03	10.0	0.000	0.28	8.5	0.000	0.28	No	
	*SRN2*	3	168.6	SNP549	2.69	4.6	0.002	−0.19	3.9	0.002	−0.19	No	
	*SRN3*	7	57.2	SNP1252	2.32	3.1	0.005	0.15	2.6	0.005	0.15	No	
PH	*PH1*	1	90.4	SNP24	2.33	3.2	0.005	1.73	3.5	0.005	1.73	No	
	*PH2*	5	132.9	SNP921	2.61	3.9	0.002	1.90	4.2	0.002	1.90	No	
	*PH3*	5	241.3	SNP1059	5.71	9.6	0.000	−2.98	10.4	0.000	−2.98	No	
LL	*LL1*	1	176.7	SNP78	2.15	0.7	0.288	−0.56	3.4	0.010	1.36	Yes	−2.42
	*LL2*	1	285.8	SNP170	5.52	7.5	0.000	−1.91	6.7	0.000	−1.91	No	
	*LL3*	2	201.8	SNP381	3.88	5.0	0.000	1.56	4.5	0.000	1.56	No	
	*LL4*	5	97.4	SNP873	3.14	4.1	0.001	1.41	3.7	0.001	1.41	No	
	*LL5*	5	236.3	SNP1058	6.48	9.4	0.000	−2.13	8.4	0.000	−2.13	No	
	*LL6*	7	171.4	SNP1382	2.75	6.0	0.001	1.70	0.2	0.551	−0.31	Yes	−5.47
LW	*LW1*	5	123.2	SNP904	2.07	1.2	0.173	−0.06	2.2	0.098	0.07	Yes	−1.21
	*LW2*	5	201.2	SNP1034	2.54	4.3	0.003	−0.11	5.5	0.003	−0.11	No	
SDW	*SDW1*	2	297.9	SNP415	3.04	3.7	0.001	0.18	5.9	0.001	0.18	No	
	*SDW2*	3	92.3	SNP444	4.57	8.5	0.000	0.27	13.4	0.000	0.27	No	
	*SDW3*	3	174.5	SNP554	5.26	10.2	0.000	−0.29	16.1	0.000	−0.29	No	
	*SDW4*	4	14.5	SNP638	2.94	3.6	0.001	0.18	5.7	0.001	0.18	No	
SFW	*SFW1*	2	247.4	SNP404	3.02	3.4	0.001	1.03	6.7	0.001	1.03	No	
	*SFW2*	7	173	SNP1384	2.10	2.2	0.008	0.83	4.3	0.008	0.83	No	
	*SFW3*	8	266.5	SNP1570	3.19	6.7	0.000	−1.43	0.1	0.776	−0.11	Yes	13.01
SPAD	*SPAD1*	1	62.2	SNP16	2.80	3.7	0.002	−1.10	4.4	0.002	−1.09	No	
	*SPAD2*	3	30.7	SNP425	2.20	5.1	0.003	1.29	0	0.899	−0.06	Yes	−23.38
	*SPAD3*	7	205.6	SNP1396	2.72	5.6	0.002	1.34	0.3	0.493	−0.30	Yes	−4.48
	*SPAD4*	9	179.9	SNP1707	3.27	4.5	0.001	1.20	5.3	0.001	1.20	No	

a *A positive value means that T877 carried the allele having an positive effect on the trait, whereas a negative value indicates that DH1M carried the favorable allele for that trait*.

b *Indicates whether the QTL showed significant QTL × environment effects*.

c *The ratio of the effects of the QTL in the two environments*.

A total of 26 identified QTLs were associated with root traits (Table 2). Four putative QTLs for CRA were detected. Only *CRA2* at SNP251 had a significant QEI, and it was detected on chromosomes 1. *CRA2* had antagonistic pleiotropic effects in the two water conditions. The T877 allele had a positive effect under WW conditions, whereas DH1M contributed a favorable allele under WS. Only one QTL associated with CRD was detected under WW and WS conditions. Sixteen QTLs were mapped for root length (three QTLs for PRL, nine QTLs for SRL, and four QTLs for CRL). Of these, six QTLs with significant QEI (*CRL1, PRL2, PRL3, SRL2, SRL7*, and *SRL9*) were detected. *CRL1* had antagonistic pleiotropic effects in the two water conditions, *PRL2* and *PRL3* were detected only under WW conditions, whereas *SRL7* and *SRL9* were detected only under WS conditions. *SRL2* had the largest contribution to SRL, 14.4% under WW conditions, whereas it only explained 1.2% of the phenotypic variation under WS conditions. Root number was mapped to five loci (three QTLs for SRN and two QTLs for CRN), all CRN-QTL were detected with significant QEI which were identified only under WW conditions. No Q × E interactions were detected for SRN-QTL. A total of 22 QTLs were identified for shoot traits, including 3, 6, 2, 4, 3, and 4 QTLs for PH, LL, LW, SDW, SFW, and SPAD, respectively. Six QTLs with significant QEI were detected (*LL1, LL6, LW1, SFW3, SPAD2*, and *SPAD3*) including five QTLs (except *SFW3*) that had antagonistic pleiotropic effects in the two watering conditions. *LL1* and *LW1* were detected only under WS conditions, and the other four were detected only under WW condition.

**Figure 5:**
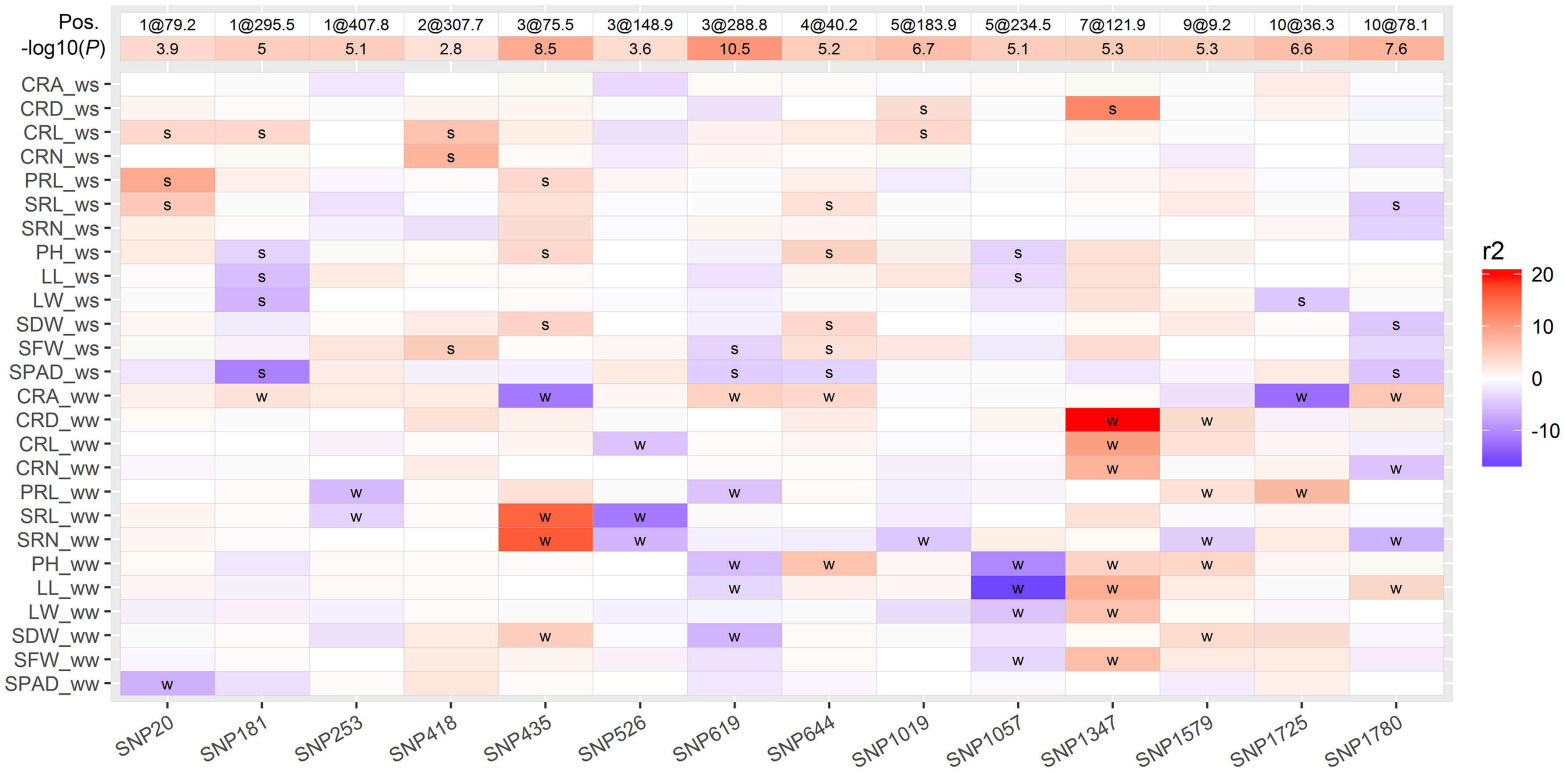
Heatmap showing genomic regions of the 14 pleiotropic QTLs detected for 13 traits. The phenotypic variation explained (*r*^2^) by each QTL is indicated by the color scale, a positive value means that T877 carried the allele having an positive effect on the trait, whereas a negative value indicates that DH1M carried the favorable allele for that trait. QTLs detected under WW and WS conditions were labeled with “w” and “s,” respectively. The position of each QTL was indicated as “chromosome@genetic positon”.

Phenotypic correlation analysis revealed that most traits were correlated with one another (Figure 2). Traits are genetically correlated due to pleiotropic QTLs or closely linked QTLs. To identify which QTLs showed pleiotropic effects, we conducted MT analysis for 13 traits under WW and WS conditions. In total, 14 regions were identified as harboring 71 putative QTLs for the 13 analyzed traits in WW and WS environments (Figure 5, Table S2). Each region consisted of 5.1 QTLs on average with a range of 2–8. SNP1347 (7@121.9 cM) consisted of eight QTLs, most of these were identified under WW conditions, and the T877 allele increased the trait value. Three loci (SNP253 at 1@407.8 cM, SNP526 at 3@288.8 cM, and SNP1579 at 9@9.2 cM) were only identified under WW conditions. SNP253 and SNP526 showed an increased effect with the DH1M allele. One locus (SNP418 at 2@307.7), which was only identified under WS conditions, showed an increased effect with the T877 allele. SNP20 (1@79.2 cM) was associated with PRL, SRL and CRL under WS conditions, and the higher value allele was from T877. SNP1057 (5@234.5 cM) was mainly associated with shoot-related traits (PH, LL, LW and SFW). Under both WW and WS, all QTLs had a positive contribution of DH1M (Figure 5).

## Discussion

Drought stress is a key abiotic stress that is responsible for the greatest crop losses all over the world (Comas et al., 2013). Previous studies have shown that root traits play critical roles in drought avoidance in maize. Such traits include small fine root diameter, long specific root length, root length density, and root angle (Lynch and Wojciechowski, 2015; Gao and Lynch, 2016). A “Steep, Cheap, and Deep” (SCD) ideotype root architecture was proposed to guide breeding strategies for new maize varieties with deeper roots and greater water acquisition from dry soil (Lynch, 2013). In addition, to adapt to water-deficit stress, maize needs to be plastic, both water availability and GEI strongly affect root architecture, such as WS could suppress crown roots (Sebastian et al., 2016) and reduced CRN could improve water acquisition under water deficit stress (Gao and Lynch, 2016), so QEI should be considered to better understand the genetic basis of root traits and response to drought (Des Marais et al., 2013). Here, ME and MT QTL analyses were conducted to identify constitutive QTLs, adaptive QTLs and pleiotropic QTLs in an RIL population.

A total of 48 QTLs were identified for 13 analyzed traits under WW and WS environments (Table 2). Most of these loci could explain <10% of the phenotypic variation, having minor effects that reflect the complexity of root traits (Burton et al., 2014; Li et al., 2015a, 2016). Several QTLs were located on the same region reported in previous studies. For example, *SRL6* at chromosome 4 (165 Mb) was close to the QTL for SRL detected in the Zheng58 × Chang7-2 RIL population, and *CRN1* and *SRL2* in bin2.06 were co-located with a QTL for total root length, axial and lateral root length, and average lateral root length (Li et al., 2016; Song et al., 2016). *SDW2* in bin3.04 was close to a QTL for shoot dry weight and root dry weight (Li et al., 2016). *SRN2* and *SRL4* in bin3.05 were co-located with a QTL for seminal root number and length, PRL and CRN (Li et al., 2015a). *SRL5*, identified on bin4.01, was also detected in the Ye478 × Wu312 population (Li et al., 2015a). There have been fewer studies on QTL mapping for root angle in maize. In this study, four root angle QTLs were identified on chromosome 1, 1, 6, and 9, and no overlap was found between our study and previous work (Giuliani et al., 2005; Omori and Mano, 2007; Hund et al., 2011; Pestsova et al., 2016), indicating that these QTLs may be novel.

Phenotypic plasticity refers to the ability of a single genotype to exhibit variable phenotypes in different environments (El-Soda et al., 2014; Song et al., 2016). Previous studies have shown that root trait plasticity in response to soil heterogeneity (water or nitrogen availability) is universal (Li et al., 2016; Kadam et al., 2017), and this plasticity can improve water or nitrogen deficit stress adaptation. When phenotypic plasticity differs among genotypes, it can be classified as a GEI (Via and Lande, 1985). A mixed model methodology with terms for QEI can be used to reveal the genetic basis of complex traits showing GEI (Boer et al., 2007). In this study, 15 QTLs with 9 traits (except CRD, SRN, PH, and SDW) were identified with significant QEI (Table 2). The additive effects of QTL with QEI across environments includes antagonistic pleiotropy (AP), conditional neutrality (CN), and differential sensitivity (DS; Des Marais et al., 2013). The term AP is used to describe a QTL with opposite effects in different environments. Considering antagonistic fitness effects in breeding programs is crucial for selection of the favored trait values in the same direction in specific environment (Rose, 1982). Here, two QTLs (*CRA2* and *CRL1*) with antagonistic pleiotropic effects were identified. *CRA2* with the T877 allele increased CRA under WW conditions and decreased it under WS conditions, and T877 had positive effects on CRL under WS conditions through *CRL1*. A total of 13 QTLs showed signs of CN, including nine and four QTLs detected under WW and WS conditions, respectively. These four drought-adaptive QTLs (*SRL7, SRL9, LL1*, and *LW1*) could be targets for selection of maize cultivars tolerant to water deficit.

In this study, strong correlations were observed among root traits and shoot traits, which is consistent with previous research (El-Soda et al., 2014; Li et al., 2015a; Kadam et al., 2017). These correlations are the result of either genetic linkage or pleiotropy (Wagner and Zhang, 2011), and the joint analysis of multiple traits (MT) could help differentiate whether these correlations are due to pleiotropic QTLs or closely linked QTLs (Jiang and Zeng, 1995). Here, 14 pleiotropic QTLs were identified for 13 traits, and each region consisted of 5.1 QTLs (Figure 5). This information could help us to design rational breeding schemes to maximize the role of each trait in marker-assisted breeding (MAS) programs. For example, SNP20 (1@79.2 cM), which was associated with length of crown roots, PR and SRs, might contribute to increased root length under WS conditions, SNP418 (2@307.7 cM) and SNP1347 might help to improve crown root architecture under both WS and WW conditions. QTLs with synergistic alleles from different parent for different traits should be used cautiously. For example, rooting depth is positively correlated with greater acquisition of water from deep soil, and deeper rooting is determined by a combination of root angle and root length. At SNP435 (3@75.5 cM), the DH1M allele contributed most to increased root angle, whereas the trait-value-enhancing effects for SR length and number was from T877. In contrast, the T877 allele contributed a greater effect for root angle at SNP619 (3@288.8 cM), whereas the favorable allele for PRL was from DH1M. These QTLs should be selectively applied for MAS breeding to improve drought adaptability in maize.

## Author contributions

PL, YZ, ZY, and CX: Conceived the experiment and made the revision of the manuscript; YZ, SY, TP, HF, and DH: Performed the research; YZ, PZ, TP, JW, and SY: Collected data; PL, YZ, and YX analyzed the data and wrote the manuscript; All authors reviewed and approved this submission.

### Conflict of interest statement

The authors declare that the research was conducted in the absence of any commercial or financial relationships that could be construed as a potential conflict of interest.
